# Testing the Identity Disruption Model among Adolescents: Pathways Connecting Adverse Childhood Experiences to Body Dissatisfaction

**DOI:** 10.1007/s10964-022-01683-5

**Published:** 2022-10-15

**Authors:** Lenny R. Vartanian, Kate Nicholls, Jasmine Fardouly

**Affiliations:** 1 School of Psychology, UNSW Sydney, Sydney, NSW Australia; 2 Centre for Emotional Health, Macquarie University, Sydney, NSW Australia

**Keywords:** Early childhood adversity, Self-concept clarity, Body dissatisfaction, Adolescents

## Abstract

The Identity Disruption Model posits that early adversity is associated with lower self-concept clarity, which in turn increases vulnerability to sociocultural appearance factors and body dissatisfaction, but this model has not previously been tested among adolescents. Testing the model during adolescence is critical because this is a key point of development of both identity and body dissatisfaction. This paper presents two studies with adolescents recruited through social media (Study 1: *n* = 213; 78% female; mean age = 15.7 years, *SD* = 1.14) and from high schools (Study 2; *n* = 228; 43% female; mean age = 13.8 years, *SD* = 1.15). In both studies, self-reported early adversity was associated with lower self-concept clarity; lower self-concept clarity was associated with greater internalization of appearance ideals and more frequent appearance comparisons; and internalization and appearance comparisons were associated with greater body dissatisfaction. This research builds on previous sociocultural models of body dissatisfaction by pointing to processes that occur early in life that could be potential targets of intervention and prevention efforts.

## Introduction

Body image is a significant concern for young people. For example, a national survey of young people in Australia consistently finds that body image is among the top concerns for youth aged 15 to 19. In 2020, 45.9% of girls and 15.7% of boys indicated that they were *very concerned* or *extremely concerned* about their body image (Tiller et al., 2021). Importantly, body dissatisfaction is associated with a range of negative psychological outcomes (including low self-esteem and depression; Paxton et al., 2006) and is one of the most robust modifiable risk factors for the development of clinical eating disorders (Jacobi et al., 2004). Eating disorders themselves are associated with heightened morbidity and mortality (e.g., Arcelus et al., 2011; Swinbourne & Touyz, 2007). Given the severity, scope, and impact of these problems, the current research sought to contribute to a better understanding of the factors associated with risk and resilience in the development of body dissatisfaction.

The most prominent models explaining body dissatisfaction center on sociocultural pressures related to appearance. For example, the Tripartite Influence Model (e.g., Thompson et al., 1999) suggests that two main pathways leading to the development of body dissatisfaction are the internalization of societal appearance ideals (i.e., the extent to which individuals take on the societal norms as personally meaningful beliefs) and appearance-based social comparisons (i.e., the extent to which individuals compare their own appearance to someone else’s appearance). There is substantial empirical support for the relevance of these two factors in correlational (e.g., Keery et al., 2004), longitudinal (Rodgers et al., 2015), and experimental (e.g., Dittmar & Howard, 2004; Tiggemann & McGill, 2004) research. These sociocultural models have been highly influential in the field, providing valuable insights into the factors that contribute to body dissatisfaction. What is missing from these models, however, is an indication of why some people are more likely to internalize societal norms and why some people are more likely to make appearance-based comparisons than are others. Identifying early risk factors is an important part of being able to intervene and prevent the development of body dissatisfaction.

A known early risk factor for body dissatisfaction and disordered eating is early childhood adversity, which can include exposure to a variety of negative circumstances early in life. Early adversity is associated with a range of negative mental and physical health outcomes, including depression, substance abuse, and heart disease (e.g., Taylor et al., 2011). In the context of disordered eating, most of the research has focused on childhood abuse. For example, a meta-analysis found that individuals who had been sexually abused in childhood had higher levels of eating pathology than did individuals who had not been sexually abused (Smolak & Murnen, 2002). Prospective studies have also shown that experiencing childhood maltreatment predicted the occurrence of eating disorders and disordered eating later in life (Johnson et al., 2002). Other studies have conceptualized early adversity more broadly (including adverse family environments), also showing elevated risk of disordered eating (e.g., Kinzl et al., 1994; Smyth et al., 2008). In fact, there is some evidence that adverse family environments (including factors such as neglect, conflict, and lack of support) have a stronger association with eating disorders than does childhood sexual abuse (Rind et al., 1998). Thus, a broad range of adverse experiences appear to be relevant in the context of disordered eating.

Although previous research has established a connection between early adversity and later body dissatisfaction and disordered eating, less is known about why or how early adverse experiences impact body dissatisfaction and disordered eating. The Identity Disruption Model (Vartanian & Hayward 2017; Vartanian et al., 2018) was developed in an attempt fill this gap in the literature by explaining how negative early life experiences could be connected to later body dissatisfaction and disordered eating. The Identity Disruption Model posits that negative early life experiences disrupt normal identity development, resulting in a less clearly defined sense of self. Early adversity might disrupt the sense of self because these early experiences are invalidating, or perhaps because individuals are deprived of experiences (such as positive interactions with caregivers) that contribute to identity development (e.g., Carlson et al., 1997). Individuals who lack a clear sense of self seek external sources to help define themselves (Campbell, 1990). Given the potency of appearance ideals in some societies, cultural ideals of attractiveness can provide an external source by which people can define themselves. Indeed, internalizing these cultural ideals is related to a greater tendency to define one’s self in terms of one’s physical appearance (Vartanian, Hayward, & Carter, in press). Thus, individuals who lack a clear sense of their own identity should be more susceptible to sociocultural factors (i.e., internalization and appearance comparisons), and consequently more likely to develop body dissatisfaction and disordered eating.

A number of studies have provided support for the Identity Disruption Model, showing that early adversity is associated with low self-concept clarity, low self-concept clarity is associated with greater internalization and appearance comparisons, which are in turn associated with greater body dissatisfaction and disordered eating (e.g., Vartanian et al., 2016, 2018; Vartanian & Hayward, 2020). These associations have been found whether early adversity is operationalized in terms of abuse experiences (e.g., sexual abuse, physical abuse) or in terms of disruptive family environments (Vartanian et al., 2018). Although consistent evidence has been found in support for the Identity Disruption Model, almost all of the studies assessing the various components of the model have included samples of young adults. The only study to date to assess components of this model in adolescents is a study that found a negative correlation between self-concept clarity and internalization of cultural appearance norms among adolescent boys (Humphreys & Paxton, 2004). It is important to examine the core components of this model among adolescents because adolescence is a developmental period during which individuals begin to form their own identities (Kroger et al., 2010), and is also a period during which internalization of the thin ideal, body-related comparison, and body dissatisfaction are likely to emerge (e.g., Rapee et al., 2019; Sands & Wardle, 2003; Schutz et al., 2002).

Another important consideration is the potential for gender differences in the constructs and pathways outlined in the Identity Disruption Model. It is fairly well-established that boys tend to show lower levels of body dissatisfaction than do girls (Prnjak et al., 2021), and there is also evidence of gender differences in internalization of cultural ideals and appearance-based comparisons (e.g., Palmeroni et al., 2021). However, there is some evidence (at least among adults) that the associations among variables in the Identity Disruption Model do not differ for men and for women (Vartanian et al., 2018). Given the lack of research on these processes among adolescents, it is worth exploring the Identity Disruption Model among both boys and girls.

## The Present Research

The Identity Disruption Model posits that early adversity is associated with lower self-concept clarity, which in turn increases vulnerability to sociocultural appearance factors and body dissatisfaction, but this model has not previously been tested among adolescents. The aim of the current research was to test the Identity Disruption Model among two separate samples of adolescents. In Study 1, adolescents were recruited through social media and completed measures of early adversity, self-concept clarity, internalization of cultural appearance norms, appearance comparisons, and body dissatisfaction. In Study 2, adolescents were recruited from schools and completed the same measures as in Study 1, except that the appearance comparison measure was specifically oriented toward comparisons on social media. Following from the Identity Disruption Model, it was predicted that early adversity would be associated with lower self-concept clarity, that lower self-concept clarity would be associated with greater internalization and appearance-comparison tendency, which in turn would be associated with greater body dissatisfaction. No firm predictions were made about any gender differences in the associations among variables in the model.

## Study 1

### Method

#### Participants

A total of 220 participants, aged 13–17 years, and based in Australia were recruited via advertisements placed on Facebook and Instagram. All participants received a $10 gift voucher for completing the survey. Participants who responded incorrectly to a data quality check (*n* = 3) or failed to complete the survey (*n* = 4) were excluded from the data, leaving 213 participants for analysis. The sample was comprised of 166 individuals who identified as female, 40 who identified as male, 5 who identified as non-binary/third gender, and 2 who preferred not to answer the question about gender. The mean age was 15.69 years (*SD* = 1.14), and the mean body-mass index (BMI) was 21.67 kg/m^2^ (*SD* = 4.16). In terms of ethnicity, 61.5% self-identified as Caucasian, 28.6% as Asian, 2.3% as Middle Eastern, and 7.1% as other/prefer not to say. This study was approved by the university’s ethics committee.

#### Materials and procedure

Participants signed up for a research study investigating adolescent health and wellbeing, completed entirely online. Because participants were under 18, written parental consent was obtained, and confirmed via phone. Participants also gave written consent prior to commencing the survey. Participants completed the following measures in a random order, in addition to other measures that were unrelated to the core components of the Identity Disruption Model and thus not included in the present research.

##### Early adversity

A single item from the Risky Families Questionnaire (RFQ; Taylor et al., 2004) was used to assess participants’ experience of early adversity. A single item was used due to concerns that the full RFQ could be too confronting given participants’ age and the fact that the study was completed entirely online. In this study, participants were asked: “Would you say that the household you grew up in was chaotic and disorganized?” Data from previous studies indicate that this single item correlates strongly with the full RFQ (around *r* = 0.80) and that the correlations between the single item and various outcome variables are similar in magnitude to the correlations with the full RFQ (Hayward et al., 2020; Vartanian et al., 2018). This question was answered on a 5-point scale (0 = *Not at all*, 4 = *Very often*) with higher scores reflecting greater adversity.

##### Self-concept clarity

The Self-Concept Clarity Scale (SCCS; Campbell et al., 1996) is a 12-item measure assessing the degree to which participants’ beliefs about themselves are clear and stable (e.g., “In general, I have a clear sense of who I am and what I am”). Each item is rated on a 7-point scale (1 = *Strongly disagree*, 7 = *Strongly agree*). After reverse-coding relevant items, higher mean scores indicate greater self-concept clarity. Good construct and criterion validity have been demonstrated for this measure (Campbell et al., 1996), and the measure has previously been used with adolescent samples (e.g., van Dijk et al., 2014). In the current study, Cronbach’s alpha was 0.86.

##### Internalization of societal appearance ideals

The internalization scale of the sociocultural attitudes towards appearance questionnaire (SATAQ-4R; Schaefer et al., 2017) was used to assess the degree to which participants had internalized societal ideals of physical appearance. The scale includes three subscales, with four items assessing a thin ideal, four items assessing a muscular ideal, and six items assessing a general attractiveness ideal. All items are rated on a 5-point scale (1 = *Definitely disagree*, 5 = *Definitely agree*). Separate mean scores were created for each subscale, with higher scores indicating greater internalization of societal ideals. Reliability and validity of the measure have been demonstrated in a range of samples, including adolescents (e.g., Palmeroni et al., 2021; Shaefer et al., 2017). All subscales showed good internal consistency in the current study (thin ideal, α = 0.84; muscular ideal, α = 0.91; general attractiveness ideal, α = 0.86).

##### Appearance comparisons

The Physical Appearance Comparisons Scale (PACS-R; Schaefer & Thompson, 2014) was used to assess the degree to which participants compare their own appearance to the appearance of others (e.g., “When I’m with a group of friends, I compare my body size to the body size of others”). The scale consists of 11 items, each of which is rated on a 5-point scale (0 = *Never*, 4 = *Always*) with higher mean scores indicating a greater tendency to make appearance comparisons. The measure has demonstrated good reliability and validity (Schaefer & Thompson, 2014), and has been used with adolescent samples previously (e.g., Palmeroni et al., 2021). Internal consistency was excellent in the current study (α = 0.96).

##### Body dissatisfaction

Participants completed the weight and shape concerns subscales of the Eating Disorder Examination Questionnaire (EDE-Q; Fairburn & Beglin, 1994). The shape concern scale is comprised of eight items (e.g., “How dissatisfied have you been with your shape?”), and the weight concern scale includes four items (e.g., “How dissatisfied have you been with your weight?”). A slight change of wording was used to tailor the items to participant gender (e.g., desire for a totally flat/six-pack stomach; Griffiths et al., 2015). One item (“Has thinking about shape or weight made it more difficult to concentrate on things you are doing?”) is proposed to load onto both weight and shape concerns. In order to maintain distinct item sets for the two subscales, and following the approach used in previous research (e.g., Vartanian et al., 2014, 2018), this item was only included in the shape concerns subscale. Both subscales refer to the last 28 days and are scored using a 7-point scale (0 = *Not at all/No days*, 6 = *Every day/Markedly*) with higher scores indicating greater concern for shape concerns and weight concerns, respectively. The measure has been shown to be reliable and valid (e.g., Mond et al., 2004), and has been used with adolescent samples previously (e.g., White et al., 2014). In the current study, internal consistency was good for girls (shape concerns, α = 0.92; weight concerns, α = 0.86) but was lower for boys (shape concerns, α = 0.64; weight concerns, α = 0.57).

Participants were also asked to complete the appearance and weight subscales of the Body Esteem Scale for Adolescents and Adults (BESAA; Mendelson et al., 2001). This scale includes 10 items for appearance esteem (e.g., “I’m pretty happy about the way I look”) and 8 items for weight esteem (e.g., “I am satisfied with my weight”). The two measures are rated on a 5-point scale (1 = *Never*, 5 = *Always*) with higher scores on each indicating more positive feelings about one’s body. The BESAA was designed to be used with adolescent samples, and has shown good reliability and validity (Mendelson et al., 2001). In the current study, internal consistency was excellent (appearance esteem, α = 0.90; weight esteem, α = 0.90).

#### Statistical analyses

There were no missing data in Study 1. Prior to conducting the main analyses, the data were screened for normality and the presence of univariate and multivariate outliers using SPSS. All variables were normally distributed except for the muscular and general attractiveness ideals subscales of the SATAQ-4R, and the weight concerns subscale of the EDE-Q. Transforming these variables improved the normality of the distribution, but had no impact on the results, and thus the analyses reported below are based on the untransformed values. One multivariate outlier was identified. Removing this participant had no impact on the pattern of results, so the analyses reported below include the full sample.

Bivariate correlations were first conducted between all variables of interest. (Descriptive statistics and bivariate correlations are presented separately for boys and girls in Appendix 5 and Appendix 6.) The primary analysis examined a structural equation model in which the single-item measure of early adversity predicted self-concept clarity; self-concept clarity predicted internalization (a latent factor reflecting the thin, muscular, and general attractiveness ideals) and appearance comparisons; and internalization and appearance comparisons predicted body dissatisfaction (a latent factor reflecting weight and shape concerns, and appearance and weight esteem). Internalization and appearance comparisons were free to covary. The model was initially constructed to include the core pathways specified in the Identity Disruption Model, but because many of the variables are known to be correlated with one another, modification indices were also examined to identify additional covariances that could be added to the model. The model was tested using AMOS version 26. Good model fit is typically indicated by a non-significant *χ*^2^ test (although this test is often significant with a large sample), RMSEA close to or under 0.06 with an upper 90% confidence interval (HI90) close to 0.08, SRMR close to 0.08, and a comparative fit index (CFI) and Tucker-Lewis index (TLI) close to 0.95 (Hu & Bentler, 1999). Multigroup structural equation modeling was used to determine whether the magnitude of the hypothesized paths in the model differed between girls and boys. Each of the paths in the model (one by one) was constrained to be equal between the groups, and the constrained paths were compared to the unconstrained paths. A non-significant *χ*^2^ test indicates no gender difference in the magnitude of the specific path. Because the sample of boys was relatively small, these multigroup results should be interpreted with caution.

### Results

#### Bivariate correlations

All bivariate correlations are presented in Table 1. Of note, the single-item measure of early adversity was negatively correlated with self-concept clarity. Self-concept clarity was negatively correlated with internalization of the thin and general attractiveness ideal (but not muscular ideal); negatively correlated with appearance comparisons and shape and weight concerns; and positively correlated with appearance and weight esteem. Internalization of the thin and general attractiveness ideal (but not the muscular ideal) and appearance comparisons were all positively correlated with shape and weight concerns, and negatively correlated with appearance and weight esteem. Early adversity was correlated with only one of the four measures of body dissatisfaction (appearance esteem) in the bivariate correlations.

**Table 1:** Bivariate Correlations between all Variables of Interest (Study 1)

	1	2	3	4	5	6	7	8	9	10
1. Early adversity	--									
2. Self-concept clarity	−0.21**	--								
3. Internalization - Thin	0.09	−0.32***	--							
4. Internalization - Muscular	0.06	−0.02	0.05	--						
5. Internalization - General attractiveness	0.11	−0.39***	0.67***	0.10	--					
6. Appearance comparisons	0.14*	−0.43***	0.66***	0.14*	0.65***	--				
7. Shape concerns	0.10	−0.39***	0.70***	0.13	0.64***	0.82***	--			
8. Weight concerns	0.12	−0.38***	0.63***	0.08	0.57***	0.75***	0.89***	--		
9. Appearance esteem	−0.15*	0.42***	−0.62***	−0.12	−0.67***	−0.73***	−0.78***	−0.68***	--	
10. Weight esteem	−0.13	0.37***	−0.57***	−0.12	−0.50***	−0.67***	−0.77***	−0.82***	0.72***	--

*p < 0.05, **p < 0.01, ***p < 0.001

#### Structural equation model

The initial hypothesized model seemed to fit the data reasonably well, χ^2^(32, *N* = 213) = 101.71, *p* < 0.001, RMSEA = 0.10 [LO90 = 0.08, HI90 = 0.12], SRMR = 0.04, CFI = 0.95, TLI = 0.93. However, examination of the standardized regression weights indicated that the muscular ideal subscale did not load well onto the internalization factor (β = 0.09, *p* = 0.26). Given that all correlations with this subscale were non-significant (other than with appearance comparisons), the muscular ideal subscale was removed from the model, meaning that internalization reflected the thin and general attractiveness ideals only. Furthermore, based on the suggested modification indices, covariances were added between the weight concerns subscale and both the appearance and weight esteem measures, and between the internalization general measure and the appearance esteem subscale.

**Fig. 1:**
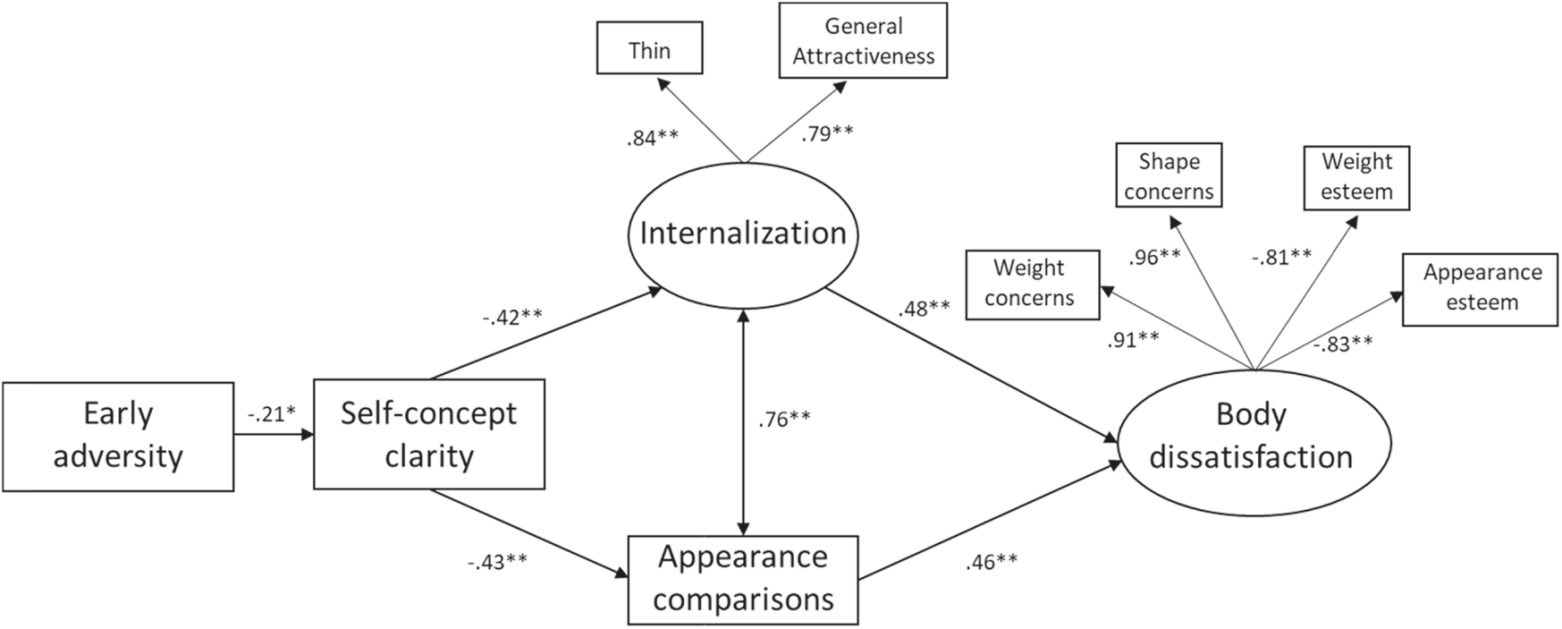
Structural Equation Model of the Identity Disruption Model (Study 1). All values are standardized regression weights. For clarity, the residuals for each variable and any covariances between them are not displayed. *p < 0.01; **p < 0.001

These changes resulted in a model with a good fit, χ^2^ (21, *N* = 213) = 29.84, *p* = 0.095, RMSEA = 0.05 [LO90 = 0.00, HI90 = 0.08], SRMR = 0.03, CFI = 0.99, TLI = 0.99 (see Fig. 1). All observed variables loaded significantly onto the relevant latent factor (*β* > 0.19*, p*s < 0.001), and all covariances were significant. All structural paths were significant. That is, greater experience of early adversity was associated with lower self-concept clarity; lower self-concept clarity was associated with greater internalization and greater appearance comparisons; and greater internalization and appearance comparisons were associated with greater body dissatisfaction.

The model explained 4% of variance in self-concept clarity, 18% of the variance in internalization, 18% of the variance in appearance comparisons, and 80% of the variance in body dissatisfaction. All indirect effects were also significant (see Table 2). Experiencing greater early adversity was indirectly associated with both greater internalization and greater appearance comparisons through lower self-concept clarity. Lower self-concept clarity was indirectly associated with greater body dissatisfaction simultaneously through greater internalization and greater appearance comparisons. Finally, early adversity was indirectly associated with greater body dissatisfaction sequentially through lower self-concept clarity and through both greater internalization and greater appearance comparisons.

**Table 2:** Standardized Coefficients and 95% Bias-Corrected Confidence Intervals for Indirect Effects (Study 1)

Pathway	Standardized Indirect effect (*β*)	95% Confidence Interval	
		Lower	Upper
Early adversity→SCC→Internalization	0.09^a^	0.02	0.17
Early adversity→SCC→Comparisons	0.09^a^	0.03	0.16
SCC→Internalization/Comparisons→BD	−0.40^a^	−0.49	−0.26
Early adversity→SCC→Internalization/Comparisons→BD	0.08^a^	0.02	0.15

*BD* Body dissatisfaction, *SCC* Self-concept clarity

^a^95% confidence interval does not cross or include 0

Multigroup analysis showed that the paths were invariant across boys and girls. However, this result needs to be interpreted with caution given the relatively small number of boys in the study.

### Discussion

The results of Study 1 broadly support the hypotheses as well as previous research on the Identity Disruption Model conducted with young adults (e.g., Vartanian et al., 2018). Although these findings support the utility of the model among adolescents, there were also some limitations to this study, including the use of a single item to capture early adversity, the relatively small sample of boys, and the low internal consistency values for the two EDE-Q subscales for boys. These issues were addressed in Study 2.

## Study 2

The primary aim of Study 2 was to replicate the findings of Study 1 with a separate sample of adolescents. The three main differences with Study 1 were as follows: First, the full version of the Risky Families Questionnaire was used rather than using just a single item to index early adversity. Second, Study 1 explored gender differences in the associations among variables, but boys were under-represented in the sample. Thus, in Study 2, an effort was made to recruit a more equal number of boys and girls. Third, the measure of appearance comparisons in Study 1 focused on overall appearance comparisons. There is growing recognition that appearance comparisons on social media can be particularly relevant to body dissatisfaction (Fardouly et al., 2017), and so this study focused on appearance comparisons on social media.

### Method

#### Participants

A total of 311 adolescents were recruited from Years 7 to 10 at two private/independent high schools in Sydney, Australia. Of those participants, 83 did not complete the variables included in the present study, which left a final sample of 228 adolescents for analysis. The final sample consisted of 98 individuals who identified as female and 130 individuals who identified as male. The mean age of participants was 13.84 years (*SD* = 1.15; range = 12–19 years) and their mean BMI was 20.21 kg/m^2^ (*SD* = 5.11). The majority of the adolescents identified as Anglo-Australian (73.2%), 15.4% identified as Eastern European, 5.7% identified as South East Asian, 3.1% identified as Mediterranean, 3.1% identified as Indigenous Australian, 2.6% identified as Pacific Islander, 3.1% identified as ‘other’ ethnicities, and 6.6% preferred not to say (participants could select more than one ethnicity to identify with). All participants went into the draw to win a $50 gift voucher for completing the survey.

#### Materials and procedure

This study was approved by the university’s human research ethics committee. Informed consent was obtained from the school principals and from one parent/primary caregiver in advance of the testing day, and the adolescents provided written consent prior to completing the questionnaires. Participants signed up for a research study investigating adolescent social media use and emotional wellbeing. Participants completed the measures used in the current study in a random order, in addition to other measures related to social media use and emotional wellbeing that were unrelated to the core components of the Identity Disruption Model and thus not included in the present research. Online surveys were completed by adolescents under exam conditions (i.e., no talking or interacting with others) in classrooms during school time.

##### Early adversity

Study 1 included only a single item asking participants about how chaotic and disorganized the household they grew up in was. To capture a broader range of negative experiences in the household, Study 2 included the full Risky Families Questionnaire (RFQ; Taylor et al., 2004). Participants responded to 11 items assessing the extent to which they had early adverse experiences with their parents and within their household (e.g., “How often would you say there was quarreling, arguing, or shouting between your parents”). Responses were made on a 5-point scale (0 = *Not at all*, 4 = *Very often*). All responses were averaged after reverse coding relevant items, with higher scores reflecting greater adversity. The RFQ has been validated against clinical interviews (Taylor et al., 2004), and has been used in adolescent samples (Miller & Chen, 2010). In the current study, Cronbach’s alpha was 0.80.

##### Self-concept clarity

As described in Study 1, the Self-Concept Clarity Scale (SCCS; Campbell et al., 1996) was used to assess the degree to which participants’ beliefs about themselves are clear and stable. Higher mean scores indicate greater self-concept clarity (α = 0.83).

##### Internalization of societal appearance ideals

The thin/low body fat and muscular/athletic internalization subscales of the sociocultural attitudes towards appearance questionnaire-4 (SATAQ-4; Schaefer et al., 2015) were used to assess the degree to which participants had internalized societal ideals of physical appearance (thin, α = 0.89; muscular, α = 0.92).

##### Appearance comparisons

Study 1 measured appearance comparisons in general. Given the research suggesting that appearance comparisons on social media are more strongly linked to body dissatisfaction than are those made in other contexts (Fardouly et al., 2017), Study 2 included an appearance comparison measure that was specifically oriented toward comparisons on social media. Similar to previous appearance comparison research with adolescents (e.g., Fardouly et al., 2020), one item was used to measure the degree to which adolescents compare their own appearance to the appearance of others on social media. Participants responded to the question “How often do you compare your physical appearance to others when using social media?” on a 5-point scale (1 = *Never*, 5 = *Very Often*).

##### Body dissatisfaction

The EDE-Q shape and weight concern scale demonstrated low internal consistencies for boys in Study 1 and were therefore not included in Study 2. Body dissatisfaction was measured with the appearance esteem subscale of the Body Esteem Scale for Adolescents and Adults (BESAA; Mendelson et al., 2001). To be consistent with the main outcome in Study 1, the measure was reverse coded so that higher scores represented greater body dissatisfaction (α = 0.86).

#### Statistical analyses

There were some missing data points for individual variables (<1% of total responses), however Little’s MCAR test indicated that these data were missing completely at random (χ^2^(472) = 359.86, *p* = 1.00). Prior to conducting the main analyses, the data were screened for normality and the presence of univariate and multivariate outliers. All variables were normally distributed except for the RFQ and the appearance comparison measure. Transforming these variables improved the normality of the distribution, but had no impact on the results, and thus the analysis reported below are based on the untransformed values. There was one individual who was identified as a univariate outlier and multivariate outlier. Removing this participant from the analyses had no impact on the pattern of results, so the analyses reported below include the full sample.

Bivariate correlations were first conducted between all variables of interest. (Descriptive statistics and bivariate correlations are presented separately for boys and girls in Appendix 7 and Appendix 8.) The primary analysis examined a structural equation model in which early adversity predicted self-concept clarity; self-concept clarity predicted internalization (a latent factor reflecting the thin and muscular ideals) and appearance comparisons; and internalization and appearance comparisons predicted body dissatisfaction. Internalization and appearance comparisons were free to covary. Multigroup structural equation modeling was used to determine whether the magnitude of the hypothesized paths in the model differed between girls and boys. As in Study 1, each of the paths in the model (one by one) was constrained to be equal between the groups, and the constrained paths were compared to the unconstrained paths. A non-significant χ^2^ test indicates no gender difference in the magnitude of the specific path.

### Results

#### Bivariate correlations

All bivariate correlations between the variables included in the study are displayed in Table 3. The early adversity measure was negatively correlated with self-concept clarity, and positively correlated with thin-ideal internalization and with body dissatisfaction. Self-concept clarity was negatively correlated with internalization (both thin and muscular ideal), appearance comparisons, and body dissatisfaction. Internalization (both thin and muscular ideal) and appearance comparisons were positively correlated with one another and with body dissatisfaction. Early adversity was positively correlated with body dissatisfaction.

**Table 3:** Bivariate Correlations between all Study Variables (Study 2)

	1	2	3	4	5	6
1. Early adversity	--					
2. Self-concept clarity	−0.35***	--				
3. Internalization - Thin	0.14*	−0.43***	--			
4. Internalization - Muscular	0.02	−0.23***	0.62***	--		
5. Appearance comparisons	0.07	−0.42***	0.60***	0.41***	--	
6. Body dissatisfaction	0.30***	−0.51***	0.58***	0.31***	0.58***	--

*p < 0.05, ***p < 0.001

#### Structural equation model

The initially hypothesized model did not fit the data particularly well, χ^2^(7, *N* = 228) = 39.54, *p* < 0.001, RMSEA = 0.14 [LO90 = 0.10, HI90 = 0.19], SRMR = 0.07, CFI = 0.93, TLI = 0.84. In line with the suggested modification indices and with previous research (e.g., Vartanian et al., 2018), a structural path was added from early adversity to body dissatisfaction. This change resulted in a model with acceptable fit, χ^2^(6, *N* = 228) = 17.80, *p* = 0.001, RMSEA = 0.09 [LO90 = 0.05, HI90 = 0.15], SRMR = 0.04, CFI = 0.97, TLI = 0.93.

**Fig. 2:**
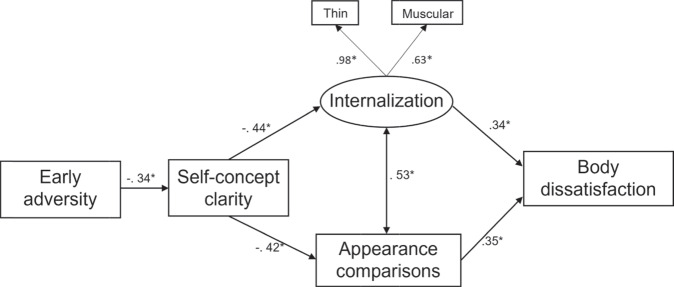
Structural Equation Model of the Identity Disruption Model (Study 2). All values are standardized regression weights. For clarity, the residuals of each variable, and the structural path from early adversity to body dissatisfaction are not displayed. *p < 0.001

All structural paths were significant (*p*s < 0.001; see Fig. 2). That is, greater experience of early adversity was associated with lower self-concept clarity, and low self-concept clarity was associated with greater internalization and greater appearance comparisons. Greater internalization and appearance comparisons were both associated with greater body dissatisfaction. Greater experience of early adversity was also directly associated with greater body dissatisfaction.

The model explained 12% of variance in self-concept clarity, 19% of variance in internalization, 18% of variance in appearance comparisons, and 48% of variance in body dissatisfaction. All indirect effects were also significant (see Table 4). Experiencing early adversity was indirectly associated with both greater internalization and greater appearance comparisons via lower self-concept clarity. Low self-concept clarity was indirectly associated with greater body dissatisfaction simultaneously through greater internalization and greater appearance comparisons. Finally, early adversity was indirectly associated with greater body dissatisfaction sequentially through lower self-concept clarity, and through both greater internalization and greater appearance comparisons.

**Table 4:** Standardized Coefficients and 95% Bias-Corrected Confidence Intervals for Indirect Effects (Study 2)

Pathway	Standardized Indirect effect (*β*)	95% Confidence Interval	
		Lower	Upper
Early adversity→SCC→Internalization	0.15^a^	0.10	0.23
Early adversity→SCC→Comparisons	0.15^a^	0.10	0.20
SCC→Internalization/Comparisons→BD	−0.30^a^	−0.39	−0.21
Early adversity→SCC→Internalization/Comparisons→BD	0.10^a^	0.07	0.15

*BD* Body dissatisfaction, *SCC* Self-concept clarity

^a^95% confidence interval does not cross or include 0

Multigroup analysis showed that only one of the paths was significantly different for boys and girls. The path between appearance comparisons and body dissatisfaction was stronger for girls (*β* = 0.43, *p* < 0.001) than it was for boys (*β* = 0.19, *p* = 0.044), χ^2^Δ= 4.01, *p* = 0.045, but the path was still significant for both groups.

### Discussion

The results of Study 2 are consistent with those of Study 1 and with the broader literature. By using the full version of the Risky Families Questionnaire (compared to the single item used in Study 1), this study provided a more robust test of the connection between early adversity and self-concept clarity. This study also provided a more robust test of potential gender differences in the associations among the variables in the model, and found that the model was stable across boys and girls. Finally, it is noteworthy that the model held when focusing specifically on appearance comparisons made on social media rather than appearance comparisons in general. Overall, the results of this study provide further support for the Identity Disruption Model of body dissatisfaction.

## General Discussion

Body dissatisfaction is a significant concern among adolescent girls and boys, and thus it is important to identity risk factors that can lead to the development of body dissatisfaction. The Identity Disruption Model (Vartanian et al., 2018; Vartanian & Hayward 2017) was developed to explain how early risk factors (childhood adversity, low self-concept clarity) can explain why some people are more susceptible to sociocultural appearance pressures than are others. This model extends pervious sociocultural models of body dissatisfaction (such as the Tripartite Influence Model; Thompson et al., 1999) by focusing on earlier risk factors. Although the model has garnered empirical support in the literature, the model had not previously been tested among adolescents. Testing the model among adolescents is important because adolescence is the developmental period during which individuals begin to form their own identities (Kroger et al., 2010), and is also a period during which internalization of the thin ideal, body-related comparison, and body dissatisfaction are likely to emerge (e.g., Sands & Wardle, 2003; Schutz et al., 2002). Thus, the aim of the present studies was to provide evidence for the utility of the Identity Disruption Model in predicting body dissatisfaction among adolescents.

Across two separate samples of adolescents with varying ages (the Study 2 sample had a mean age that was almost 2 years younger than that of the Study 1 sample), results showed that early adversity predicted lower self-concept clarity, lower self-concept clarity was associated with greater internalization of appearance ideals and greater appearance comparisons, which were in turn associated with greater body dissatisfaction. These findings are consistent with other research demonstrating these pathways among young adults (e.g., Vartanian et al., 2018), and show that the model has relevance among adolescents as well. By demonstrating that the Identity Disruption Model predicts body dissatisfaction among adolescents, this research points to a potential target of early prevention efforts to reduce the burden of body dissatisfaction and disordered eating. Reducing the prevalence of early adversity should of course be of primary concern, but building resilience among those who do experience early adversity is also an important objective, and self-concept clarity might be a useful target in that respect.

A notable strength of the present study was the inclusion of boys and girls in both samples. The vast majority of research on body image has focused on girls and young women. Although the prevalence of body dissatisfaction is generally higher among girls than among boys (Tiller et al., 2021), there is growing recognition that body image issues are significant concerns for boys as well (Nagata et al., 2021; Pope et al., 2000). It is important for research to examine whether the processes linked to body dissatisfaction are similar for boys and girls. Most studies testing the Identity Disruption Model have been limited to female samples, but one study with adolescent boys did find an association between self-concept clarity and internalization (Humphreys & Thompson, 2004), and another study found that the full Identity Disruption Model held for women and for men alike (Vartanian et al., 2018). The current studies add to this evidence base by demonstrating that the paths in the model did not differ for adolescent girls and boys (except that the path between comparisons and body dissatisfaction in Study 2 was weaker, but still significant, for boys than it was for girls). These findings suggest that experiences with early adversity are just as likely to contribute to low self-concept clarity and increase the risk of body dissatisfaction for boys and girls. These results also suggest that interventions based on the Identity Disruption Model could be relevant to both genders, making them easier to implement on a broad scale.

Another noteworthy finding from this research is the fact that the Identity Disruption Model was relevant to both general appearance comparisons (replicating previous research) and appearance comparisons on social media. This adds to the generalizability of the model, but also indicates the importance of social media as a forum for engaging in appearance comparisons among adolescents. Adolescents spend around two hours on social media each day (e.g., Fardouly et al., 2022; Statistica, 2019), and primarily engage with appearance-based media (e.g., Instagram, TikTok), providing them with ample opportunities to engage in appearance comparisons. If low self-concept clarity increases the likelihood that they will compare themselves to the people they see on social media, then this can accumulate overtime, increasing the risk of body dissatisfaction. Indeed, experimental research suggests that those low in self-concept clarity are more likely to make appearance comparisons when viewing thin-ideal social media images, which in turn increases their state body dissatisfaction (Carter & Vartanian, 2022). Thus, it may be particularly important for adolescents with low self-concept clarity to reduce their opportunities to make comparisons to attractive others on social media by unfollowing accounts that posts such content (e.g., beauty or fitness influencers) in order to protect their body image.

There are some limitations to the present research that point to opportunities for future research. First, the data from the current studies are cross-sectional, limiting any inferences that can be drawn about causal associations or temporal sequencing among the variables in the model. Longitudinal studies, particularly among children and early-adolescents, would be important to establish the developmental trajectory of the proposed pathways. Experimental studies (e.g., studies manipulating the level of self-concept clarity) could be useful for examining the causal impact of self-concept clarity on sociocultural factors (such as the likelihood of engaging in appearance-based social comparisons). Studies using ecological momentary assessment could also be useful for establishing the connection between self-concept clarity and body dissatisfaction in everyday life.

Second, the measure of early adversity only captured general negative experience in the household one grew up in (and, in the case of Study 1, was limited to a single item asking about how chaotic and disorganized the household was). Previous studies have shown that the Identity Disruption Model also holds when early adversity is operationalized in terms of reports of childhood abuse (sexual abuse, physical abuse, and neglect; e.g., Hayward et al., 2020; Vartanian et al., 2018), suggesting that the findings are not limited to disruptive family environments and could apply to more severe forms of early adversity. It would be worthwhile for future research to consider other types of adversity (e.g., peer bullying, poverty) that could impact self-concept clarity and, consequently, place adolescents at risk for body dissatisfaction and disordered eating. It would also be worth exploring whether there are differences between boys and girls in how strongly different types of adversity are linked to body dissatisfaction and eating disorders. A more systematic investigation of these issues would be a valuable goal for future research.

The Identity Disruption Model can also potentially be applied to other forms of psychological maladjustment or problematic behavior. At its core, this model indicates that early adverse life experiences result in disrupted personal identity. This disrupted identity then provides a risk factor for disordered eating because the sociocultural appearance norms and pressures provide individuals with an (unhealthy) avenue for self-definition. Individuals with disrupted identity could also face difficulties in domains that are unrelated to body image (e.g., depression, substance abuse, internet addiction; Israelashvili et al., 2012; Smith et al., 1996). Indeed, a recent study found that self-concept clarity mediated the association between early adversity and symptoms of depression and anxiety (Hayward et al., 2020). Thus, the Identity Disruption Model provides a theoretical framework that could be broadly applicable for understanding psychopathology. It may well be that there are different mechanisms accounting for the connection between identity disruption and psychopathology (e.g., sociocultural appearance factors should be relevant to body dissatisfaction but not, say, to substance abuse), and future research specifically delineating the unique and/or common pathways to psychopathology would be a valuable contribution to the literature.

There are also a number of potential practical implications of the Identity Disruption Model that are worth considering. First, identifying early risk factors can indicate points of early intervention for vulnerable groups. Prevention programs that target high-risk individuals tend to elicit the largest effects (e.g., Stice & Shaw, 2004). Thus, targeting prevention programs at individuals who experience early adversity or who have low self-concept clarity could be beneficial, particularly if these issues are identified early. Second, the model also points to potential interventions, such as interventions designed to boost self-concept clarity as a means of reducing the negative impact of sociocultural appearance pressures. For example, adapting expressive-writing interventions (Lepore & Smyth, 2002) to incorporate reflection on (non-appearance-related) self-defining characteristics might help solidify the sense of self. By increasing self-concept clarity, and encouraging individuals to define themselves by means other than their appearance, such interventions could reduce the impact of sociocultural pressures and, consequently, reduce the risk of body dissatisfaction. If the Identity Disruption Model is shown to be relevant to other forms of psychopathology, then these types of interventions could have far wider benefits as well.

## Conclusion

The Identity Disruption Model was developed to help explain the connection between early adversity and body dissatisfaction. In particular, this model proposes that early adversity is associated with lower self-concept clarity, which in turn increases vulnerability to sociocultural factors (internalization, appearance comparisons) that contribute to body dissatisfaction. Previous support for the model comes from research with young adults, but testing the model during adolescence is critical because this is a key point of development of both identity and body dissatisfaction. Across two studies with adolescents, we found that low self-concept clarity was associated with greater internalization of societal appearance ideals and appearance comparisons, which were in turn associated with greater body dissatisfaction. The present studies add to the existing research by providing evidence that the Identity Disruption Model has utility in predicting body dissatisfaction among adolescent girls and boys. This research builds on previous sociocultural models of body dissatisfaction by pointing to processes that occur early in life that could be potential targets of intervention and prevention efforts.

## Appendix

Tables 5–8

**Table 5:** Bivariate Correlations Separated by Gender for all Study Variables (Study 1)

	1	2	3	4	5	6	7	8	9	10
1. Early adversity	--	−0.05	0.01	0.15	0.29	0.28	0.22	0.12	−0.31	−0.17
2. Self-concept clarity	−0.19*	--	−0.21	−0.22	−0.36*	−0.17	−0.37*	−0.26	0.31	0.19
3. Internalization - Thin	0.06	−0.26**	--	−0.02	0.42**	0.24	0.22	0.11	−0.40*	−0.16
4. Internalization - Muscular	0.07	−0.06	0.20**	--	0.31*	0.37*	0.28	0.18	−0.28	−0.22
5. Internalization - General attractiveness	0.06	−0.36***	0.63***	0.16*	--	0.60***	0.48**	0.28*	−0.70***	−0.25
6. Appearance comparisons	0.11	−0.44***	0.68***	0.22**	0.63***	--	0.73***	0.56***	−0.65***	−0.53***
7. Shape concerns	0.08	−0.36***	0.73***	0.22**	0.65***	0.82***	--	0.71***	−0.67***	−0.59***
8. Weight concerns	0.11	−0.37***	0.64***	0.16*	0.59***	0.75***	0.89***	--	−0.44**	−0.74***
9. Appearance esteem	−0.11	0.42***	−0.64***	−0.18*	−0.64***	−0.74***	−0.80***	−0.71***	--	0.57***
10. Weight esteem	−0.10	0.39***	−0.64***	−0.17*	−0.55***	−0.71***	−0.81***	−0.85***	0.74***	--

Correlations for girls are below the diagonal and those for boys are above the diagonal

**p* < 0.05, ***p* < 0.01, ****p* < 0.001

**Table 6:** Descriptive Statistics and Group Comparisons by Gender (Study 1)

	Boys	Girls			
Variable	*M* (*SD*)	*M* (*SD*)	*t*-value	*p*-value	Cohen’s *d*
Early adversity	1.45 (1.09)	1.69 (1.26)	1.10	0.275	−0.19
Self-concept clarity	4.04 (0.92)	3.45 (1.13)	3.03	0.003	0.53
Internalization - Thin	2.49 (0.89)	3.51 (1.00)	5.90	< 0.001	−1.04
Internalization - Muscular	3.43 (1.03)	2.78 (1.08)	3.43	< 0.001	0.60
Internalization - General attractiveness	3.69 (0.72)	4.20 (0.66)	4.26	< 0.001	−0.75
Appearance comparisons	1.18 (0.85)	2.15 (1.08)	6.18	< 0.001	−0.94
Shape concerns	1.71 (0.99)	3.00 (1.70)	6.36	< 0.001	−0.82
Weight concerns	1.46 (1.14)	3.02 (1.84)	6.97	< 0.001	−0.90
Appearance esteem	3.15 (0.73)	2.74 (0.84)	2.81	0.005	0.50
Weight esteem	3.41 (0.91)	3.05 (1.01)	2.07	0.040	0.37
Age	15.60 (1.17)	15.72 (1.10)	0.59	0.554	−0.10
BMI	21.57 (4.66)	21.70 (3.98)	0.18	0.871	−0.03

**Table 7:** Bivariate Correlations Separated by Gender for all Study Variables (Study 2)

	1	2	3	4	5	6
1. Early adversity	--	−0.35***	0.02	0.04	0.03	0.23**
2. Self-concept clarity	−0.32**	--	−0.20*	−0.26**	−0.35***	−0.25**
3. Internalization - Thin	0.16*	−0.36***	--	0.70***	0.31***	0.34***
4. Internalization - Muscular	−0.04	−0.14	0.65***	--	0.46***	0.26**
5. Appearance comparisons	−0.01	−0.20	0.51***	0.38***	--	0.30**
6. Body dissatisfaction	0.34**	−0.58***	0.57***	0.32**	0.60***	--

Correlations for girls are below the diagonal and those for boys are above the diagonal

**p* < 0.05, ***p* < 0.01, ****p* < 0.001

**Table 8:** Descriptive Statistics and Group Comparisons by Gender (Study 2)

Variable	Boys	Girls	*t*-value	*p*-value	Cohen’s *d*
	*M* (*SD*)	*M* (*SD*)			
Early adversity	0.88 (0.56)	1.01 (0.64)	1.61	0.109	−0.22
Self-concept clarity	5.07 (0.93)	4.25 (1.10)	6.13	<0.001	0.82
Internalization - Thin	2.06 (0.87)	3.33 (1.02)	10.09	<0.001	−1.35
Internalization - Muscular	2.56 (1.11)	2.80 (1.12)	1.68	0.094	−0.23
Appearance comparisons	1.84 (1.06)	3.44 (1.23)	10.53	<0.001	−1.41
Body dissatisfaction	2.21 (0.65)	2.87 (0.85)	6.46	<0.001	−0.90
Age	13.71 (1.12)	14.01 (1.17)	1.93	0.054	−0.26
BMI	20.43 (5.61)	19.94 (4.39)	0.70	0.486	0.10
